# Molecular Characterization of Porcine Epidemic Diarrhea Virus and Its New Genetic Classification Based on the Nucleocapsid Gene

**DOI:** 10.3390/v12080790

**Published:** 2020-07-23

**Authors:** Sung-Jae Kim, Van-Giap Nguyen, Thi-My-Le Huynh, Yong-Ho Park, Bong-Kyun Park, Hee-Chun Chung

**Affiliations:** 1 Department of Veterinary Medicine Virology Lab, College of Veterinary Medicine and Research Institute for Veterinary Science, Seoul National University, Seoul 08826, Korea; kvirus7734@gmail.com; 2 Department of Veterinary Microbiology and Infectious Diseases, Faculty of Veterinary Medicine, Vietnam National University of Agriculture, Hanoi 100000, Vietnam; nvgiap@vnua.edu.vn (V.-G.N.); huynhtmle@vnua.edu.vn (T.-M.-L.H.); 3 Department of Veterinary Microbiology, College of Veterinary Medicine and Research Institute for Veterinary Science, Seoul National University, Seoul 151-742, Korea

**Keywords:** porcine epidemic diarrhea virus, phylogenetic analysis, antigenicity, S gene, N gene

## Abstract

*Porcine epidemic diarrhea virus* (*PEDV*) causes continuous, significant damage to the swine industry worldwide. By RT-PCR-based methods, this study demonstrated the ongoing presence of *PEDV* in pigs of all ages in Korea at the average detection rate of 9.92%. By the application of Bayesian phylogenetic analysis, it was found that the nucleocapsid (N) gene of *PEDV* could evolve at similar rates to the spike (S) gene at the order of 10^−4^ substitutions/site/year. Based on branching patterns of *PEDV* strains, three main N gene-base genogroups (N1, N2, and N3) and two sub-genogroups (N3a, N3b) were proposed in this study. By analyzing the antigenic index, possible antigenic differences also emerged in both the spike and nucleocapsid proteins between the three genogroups. The antigenic indexes of genogroup N3 strains were significantly lower compared with those of genogroups N1 and N2 strains in the B-cell epitope of the nucleocapsid protein. Similarly, significantly lower antigenic indexes in some parts of the B-cell epitope sequences of the spike protein (COE, S1D, and 2C10) were also identified. *PEDV* mutants derived from genetic mutations of the S and N genes may cause severe damage to swine farms by evading established host immunities.

## 1. Introduction

*Porcine epidemic diarrhea virus* (*PEDV*) is an enveloped, single-stranded RNA virus belonging to the family *Coronaviridae*, subfamily *Coronavirinae*, genus *Alphacoronavirus,* and subgenus *Pedacovirus*. *PEDV* is one of the major pathogens causing acute enteritis disease, which is characterized by vomiting and watery diarrhea and commonly leads to high rates of mortality and morbidity in suckling piglets [1]. The disease was first reported in the UK in 1971, and the prototype virus—designated as *PEDV* CV777—was subsequently identified in Belgium [2]. Since the 1980s, *PEDV* has been widespread throughout Asia, where it has been regarded as an endemic disease for many years [3,4]. In the late 2010s, new and highly pathogenic strains were reported in China. These new strains were pathologically more critical than the classic strains, resulting in morbidities of 80–100% and mortality rates of 50–100% in infected suckling piglets [5]. In May 2013, these new highly pathogenic strains moved from China to the USA and rapidly spread across the country, massively impacting the swine industry; they affected more than 4000 farms, accounting for the death of more than 7 million piglets within the year [6,7]. Subsequently, these strains have become pandemic [8].

*PEDV* has an approximately 28 kb long genome and consists of seven open reading frames (ORF), encoding non-structural or structural proteins [9]. ORF1ab and ORF3 genes encode non-structural proteins. ORF1a codes for the large polyprotein PP1a, while ORF1b is always expressed with PP1a as the fusion protein PP1a/b through ribosomal frameshifting. PP1a and PP1a/b are further processed into 16 non-structural proteins (nsp1 to nsp16). ORF3 codes an accessory protein that is likely to be an additional non-structural protein [10]. The envelope (E), membrane (M), spike (S), and nucleocapsid (N) genes encode four major structural proteins [11]. The N protein, which is the most abundant protein in the virus particle, provides the structural basis for the helical nucleocapsid surrounding the virus genome [12,13]. The M and E proteins form a viral envelope by assembly. The M protein is the most abundant component, and the E protein is less abundant in the viral envelope. [14]. The S protein is very exposed and forms large petal-shaped spikes on the surface of the virus [15].

Among these structural proteins, the S protein plays important roles in virus infection and the induction of neutralizing antibodies [16] and shows substantial genetic diversity [11]. As a result of these features, genetic analyses based on the S gene have been commonly used to investigate *PEDV* evolution [7,8,17]. The *PEDV* has diverged into several subgroups based on the genetic diversification of the S gene. So far, two genotypes (G1 and G2) have been identified by S gene phylogenetic analysis. Each genogroup can be further divided into two subtypes, G1a/b and G2a/b, respectively [18,19]. Recently, a third subtype (G2c) was identified within the G2 genogroup [8]. The N protein is involved in several biological and immunological activities of the virus, including viral nucleolar localization, host cycle ER stress, S-phase prolongation, the inhibition of interferon-β production [20,21,22], and the induction of abundant antibodies [12,13]. Despite the significant features of the N protein, there has been less investigation on the N protein compared to the S protein. In order to better understand the evolutionary aspects of *PEDV*, this study applied multiple bioinformatic tools to investigate the genetic diversity of *PEDV*.

## 2. Materials and Methods

### 2.1. Sample Collection, PEDV Detection by PCR, and Complete Sequencing

Six hundred and seventy-two fecal samples were collected from 83 farms in 2017, and 235 fecal samples were collected from 33 farms in 2018. Rectal swabs were randomly collected from pigs of all stages (suckling, weaned, growing, finishing, gilt, sow) in swine farms dispersed throughout 9 provinces of South Korea. RNA extraction from the samples was performed using an RNA/DNA extraction kit (Invitrogen, Carlsbad, CA, USA) according to the manufacturer’s instructions, and the extracted RNA samples were stored at −70 °C. The RNA was converted into cDNA with a commercial kit (RNA to cDNA EcoDry Premix, Clontech, Otsu, Japan), following the manufacturer’s protocol. *PEDV* in the collected fecal samples was detected by PEDV-PCR [4]. Commercial PCR kits (Median diagnostics, Chuncheon-si,, Korea) were used to detect Porcine reproductive and respiratory syndrome virus (cat. NS-PRR-11), Porcine parvovirus (cat. NS-ABO-11), and Japanese encephalitis virus (cat. NS-ABO-12). The other three enteric pathogens were detected by specific primers and PCR conditions reported previously, such as Porcine deltacoronavirus [23], Transmissible gastroenteritis virus, and Porcine group A rotavirus [24].

Full-length genome sequencing was conducted with 26 overlapping primer pairs using positive samples from swine farms that were severely damaged by porcine epidemic diarrhea [25], and 7 strains (Y178, S6, S10, S12, S14, S97 and S100) were fully sequenced. Information relating to the 7 strains is provided in Table 1. The full-length genome sequences of the 7 strains were registered in GenBank (accession numbers MH891584–MH891590).

### 2.2. Genetic Analysis of Recombination

For the genetic analyses, 72 other previously registered complete genome sequences were retrieved from GenBank, and the sequences originated from Asia (China, Korea, Japan), America (USA), and Europe (Germany, Belgium) from 1978 to 2018. RDP v4.51 program [26] with 5 default algorithms (RDP, GENECONV, MaxChi, SiScan, and Bootscan) was applied to identify recombinants in the alignment of the S and N genes. The general options were “sequences are linear”, “highest acceptable *p*-value = 0.05”, and “Bonferroni correction” = true. Upon detection, new recombination-free alignments were created by the option of “save alignment with recombinant regions removed”. Four datasets with identical number of sequences (*n* = 79, seven obtained in this study) were generated for subsequent analyses: complete S gene (D1), S gene without recombinant regions (D2), complete N gene (D3), and N gene without recombinant regions (D4).

### 2.3. Bayesian Phylogenetic Analysis

BEAST package v2.6.1 [27], which is available at the CIPRES Science Gateway [28], was used to infer the phylogenetic relationships between sequences and co-estimate the substitution rates from the above-mentioned D1–D4 datasets. The genetic classification of *PEDV* based on the S gene followed previous publication [8]. For the model of nucleotide substitution, bModelTest tool [29] implemented in BEAST 2 was selected, which helps infer the most appropriate substitution model. For the molecular clock model, four models were specified: strict clock, uncorrelated lognormal and exponential relaxed-clock [30], and random local clock [31]. For tree prior, three coalescent models implemented in BEAST 2 were tested, including coalescent constant population, coalescent exponential population, and coalescent Bayesian skyline plot [32]. Each analysis was run for 100 million chains, sampling every 10,000 generations. The output log files were analyzed in Tracer v1.7.1 [33] to assess the convergence (effective sample size > 100). Path sampling analyses [34] were also performed to select the best fitting molecular clock and tree prior models for each dataset. For that analysis, the number of path steps was 100, and the length of each chain was one million iterations. The nucleotide substitution rates and phylogenetic tree of each D1–D4 dataset were inferred from the data best-fit combining models (supplementary material Tables S1 and S2). The phylogenic trees were summarized with TreeAnnotator v2.6.1 to produce the maximum clade credibility tree, which was displayed using FigTree v1.4.3.

### 2.4. p-Distance Analysis

Pairwise genetic distances (*p*-distances) within each dataset were calculated using MEGA V. 7 software [35]. The option for gaps/missing data treatment was specified as “partial deletion”. The obtained results were displayed in a frequency distribution histogram of *p*-distance. Basically, a lower genetic relationship between two sequences indicated a higher *p*-distance, and well-bounded areas with peaks in the histogram indicated the presence of different genetic clusters [36].

### 2.5. Inferring Ancestral Amino Acid Changes

The Baseml program implemented in package PAML 4.9j [37] was used to reconstruct amino acid changes on the evolutionary path of *PEDV* based on the N gene. The input tree topology for that analysis was the maximum clade credibility tree inferred by BEAST 2 under the data best-fit combining models. Nonsynonymous substitutions that occurred on the given branches of a phylogeny were annotated by the treesub program [38].

### 2.6. Amino Acids and Antigenic Index Analysis of N Gene

Amino acid sequences deduced from nucleotide sequences of the N gene were aligned and comparatively analyzed to investigate the nonsynonymous changes according to genetically distinct clusters. Subsequently, antigenic index analysis of the complete amino acid sequences of the N gene was performed to evaluate the possible antigenic variation of N proteins. The antigenic index of each amino acid was calculated by the Jameson–Wolf method [39], and the calculated indexes of each strain were compared.

### 2.7. Antigenic Index Analysis of B-Cell Epitopes in Korean PEDV Strains

Antigenic index analyses were performed to evaluate the antigenic variation of S proteins within the Korean *PEDV* strains. The antigenic index of each amino acid was calculated by the Jameson–Wolf method for 3 previously identified S protein-neutralizing epitopes, COE (within the S1 ^B^ region) [40], S1D [41], and 2C10 [42]. Subsequently, the calculated indexes of the Korean strains were compared.

## 3. Results

### 3.1. The Detection of PEDV in Korea from 2017 to 2018

**Figure 1:**
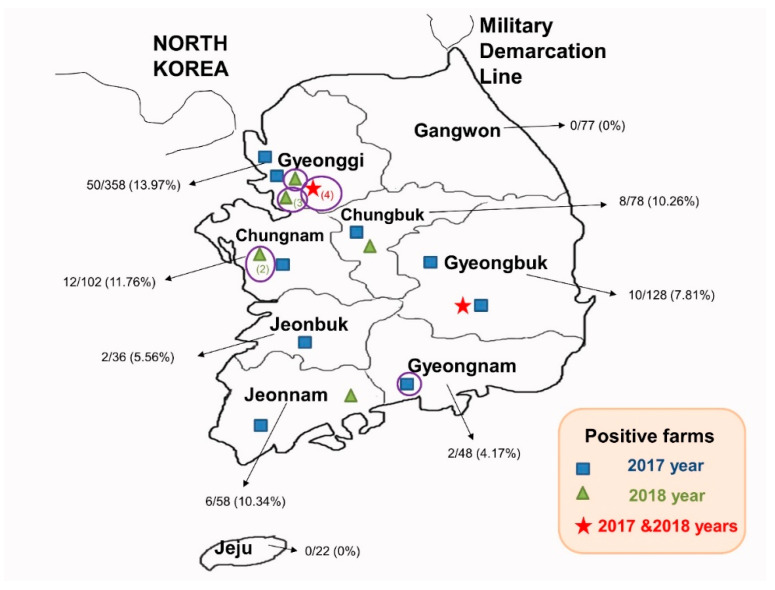
Distribution of total *PEDV*-positive swine farms in nine provinces of South Korea from 2017 to 2018. Marks (square, triangle, and star) indicated the locations of the swine farms. If the swine farms were located in the adjacent spot, the number of these swine farms was indicated in parentheses below the marks. The sample numbers (positive samples/total samples) and detection rate of each province are indicated by black arrows. The purple circles indicate the *PEDV*-positive farms where seven Korean *PEDV* strains sequenced in this study originated.

The detection rate of *PEDV* from 2017 to 2018 was 9.92% (90/907). Specifically, the positive rates in 2017 and 2018 were 8.63% (58/672) and 13.62% (32/235), respectively. The positive rate in 2018 had somewhat increased compared with that in 2017. From the detection rates of each growth stage, the highest rate was seen in the suckling stage (Table 2). Geographically, the province with a higher concentration of swine farms showed higher positive samples of *PEDV* (Figure 1).

### 3.2. Phylogenetic Analysis of Global PEDV Strains

**Figure 2:**
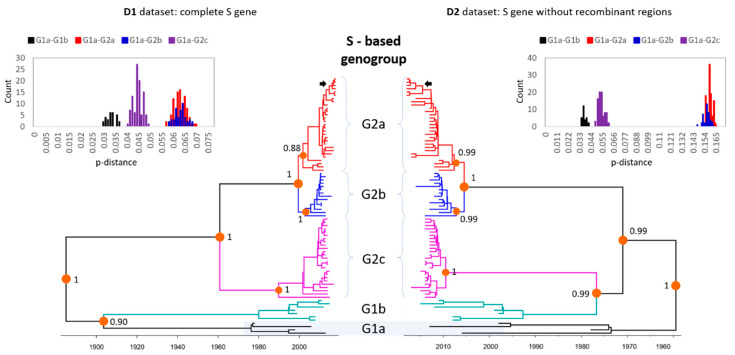
The time-scale maximum clade credibility phylogeny of global *PEDV* strains based on the S gene. The phylogenetic trees were constructed based on the S gene without removing the recombinant regions (D1) and with the recombinant regions removed (D2). The sub-genogroups were designated as G1a, G1b, G2a, G2b, and G2c. Each sub-genogroup was colored consistently between the D1 and D2 datasets. The inserted histograms of pairwise *p*-distances were between sub-genogroups G1a–G1b, G1a–G2a, G1a–G2b, and G1a–G2c. The *p*-distance is calculated by dividing the number of nucleotide differences by the total number of nucleotides compared. After removing recombinant regions, some sequences might contain large deletions. In other words, the total number of nucleotides became smaller. Thus, the *p*-distance on the right panel was larger than that on the left panel. The Korean strains identified in this study are marked by arrows.

In the phylogenetic trees inferred from the D1–D2 datasets of the S gene (Figure 2, Supplementary Figures S1 and S2), the *PEDV* strains were classified into five sub-genogroups (G1a, G1b, G2a, G2b, and G2c), which were previously designated [8]. However, the relationships between sub-genogroups differed. The D1 dataset contains the complete S gene supported for the clusters of (G1a, G1b) and (G2c, (G2a, G2b)). The D2 dataset contains the S gene without recombinant regions supported for the different clusters of (G1b, G2c) and (G2b, G2a). In both datasets, histograms of pairwise *p*-distances exhibited a discrete distribution betwen sub-genogroups. That was in agreement with the tree topologies and posterior support values at the nodes to each sub-genogroup (Figure 2). In both the D1 and D2 datasets, the seven Korean strains identified in this study (S6, S10, S12, S14, S97, S100, and Y178) were clustered within sub-genogroup G2a.

**Figure 3:**
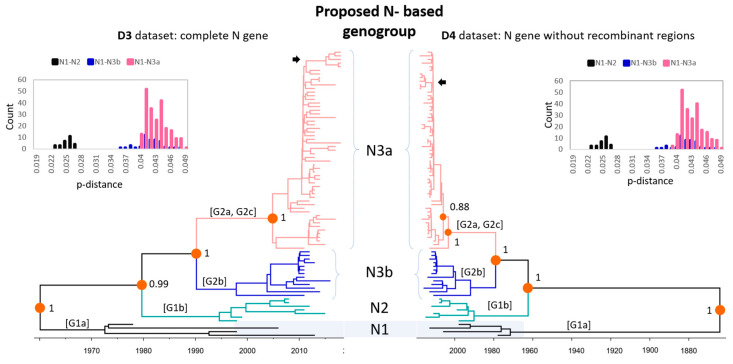
The time-scale maximum clade credibility phylogeny of global *PEDV* strains based on the nucleocapsid (N) gene. The phylogenetic trees were constructed based on the N gene without removing the recombinant regions (D3) and with the recombinant regions removed (D4). The spike (S) gene-based sub-genogroups were designated as G1a, G1b, G2a, G2b, and G2c. Each sub-genogroup was colored consistently with the D1–D4 datasets. The inserted panels are histograms of pairwise *p*-distances between geno-, sub-genogroups N1–N2 and N1–N3a/N3b. The Korean strains identified in this study are marked by arrows.

Supported by high posterior probability values (0.90–1), the phylogenetic trees inferred from the D3–D4 datasets of the N gene (Figure 3, Supplementary Figures S3 and S4) suggested that the classification of *PEDV* strains into four S gene-based sub-genogroups G1a, G1b, G2b, G2a/G2c was more reliable. In both datasets, it was observed that the S gene-based G2a and G2c were not monophyletic (pink branches). Differing from the S gene-based phylogenies (Figure 2), the Ngene-based trees with or without the elimination of recombinant regions had identical topologies of (G1a, (G1b, (G2b, G2a/G2c))). As the result, this study proposed an N-based genotyping of *PEDV* as N1, N2, N3b and N3a, which were equivalent to the S-based genotyping of G1a, G1b, G2b, and G2a/G2c, respectively. That classification was also supported by a clear bimodal distribution of genetic distance between the proposed genogroups N1–N2 and N1–N3a/N3b (inserted histograms, Figure 3). According to that scheme, the seven Korean strains identified in this study (S6, S10, S12, S14, S97, S100, and Y178) were within sub-genogroup N3a.

### 3.3. Evolutionary Rates of PEDV Genes

The estimated mean nucleotide substitutions of the S and N genes were at the order of 10^−4^ nucleotide substitutions/site/year (Table 3). The substitution rates of the complete S and N genes were not significantly different, as the 95% highest posterior density (HPD) overlapped (Table 3). Inferring from two datasets where the recombinant regions were removed, the S gene showed substantially higher substitution rates than the N gene because of non-overlapping 95% HPD (6.18 × 10^−4^–10.02 × 10^−4^ versus 2.12 × 10^−4^–4.52 × 10^−4^, respectively).

### 3.4. Amino Acids and Antigenic Index Analysis of N Gene Sequences

**Figure 4:**
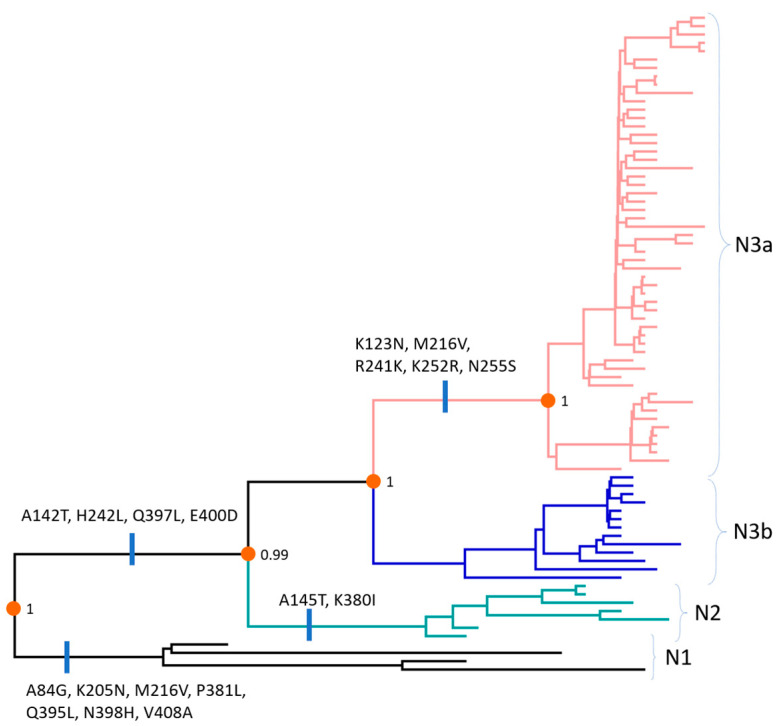
The maximum clade credibility tree based on the N gene with reconstructed nonsynonymous substitutions were mapped to the branches of the phylogeny. For clarity, posterior values were shown for main separating nodes.

Several consistent changes allowed the differentiation of the genogroups and sub-genogroups based on the N gene (Figure 4, Supplementary Figure S5). The complete list of nonsynonymous changes was given in the Supplementary Figure S5. Genogroup N1 differed from genogroups N2 and N3 by 7 nonsynonymous substitutions (A84G, K205N, M216V, P381L, Q395L, N398H, and V408A). Unique changes leading to branches (N2, (N3a, N3b)) were A142T, H242L, Q397L, and E400D. Genogroup N2 was further characterized by two nonsynonymous substitutions (A145T, K380I). Finally, the main branch leading to sub-genogroup N3a displayed five changes (K123N, M216V, R241K, K252R, and N255S).

**Figure 5:**
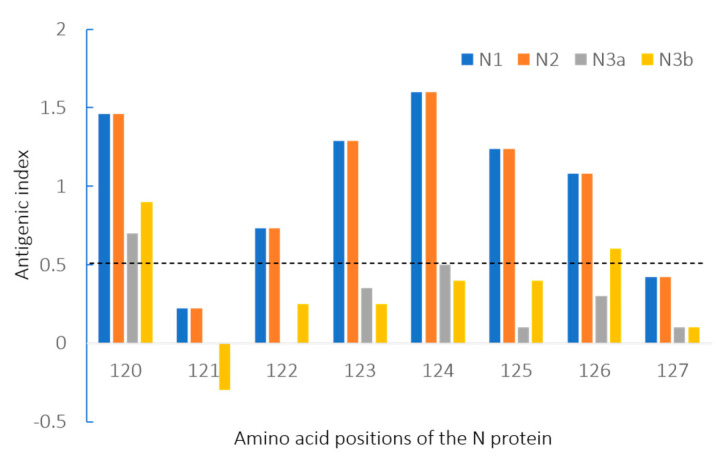
Antigenic index analysis of N gene sequences in *PEDV* strains. Genogroup N3 exhibited lower antigenic indexes, below 0.5 (cut-off value), compared with genogroups N1 and N2. This region is in a B-cell epitope sequence of the N protein, which is one of the *PEDV*-specific epitopes (amino acids 18–133 and 252–262).

In the antigenic index analysis of N gene sequences, significant reductions were found in amino acid positions 122–126 according to the genogroups (Figure 5). Genogroup N3 exhibited lower antigenic indexes, below 0.5 (the cut-off value), compared with genogroups N1 and N2. These amino acid sequences were located in a B-cell epitope sequence of the N protein, which is one of the *PEDV*-specific epitopes (amino acids 18–133 and 252–262) previously identified [43].

### 3.5. Antigenic Index Analysis of S Protein B-Cell Epitopes in Korean PEDV Strains

**Figure 6:**
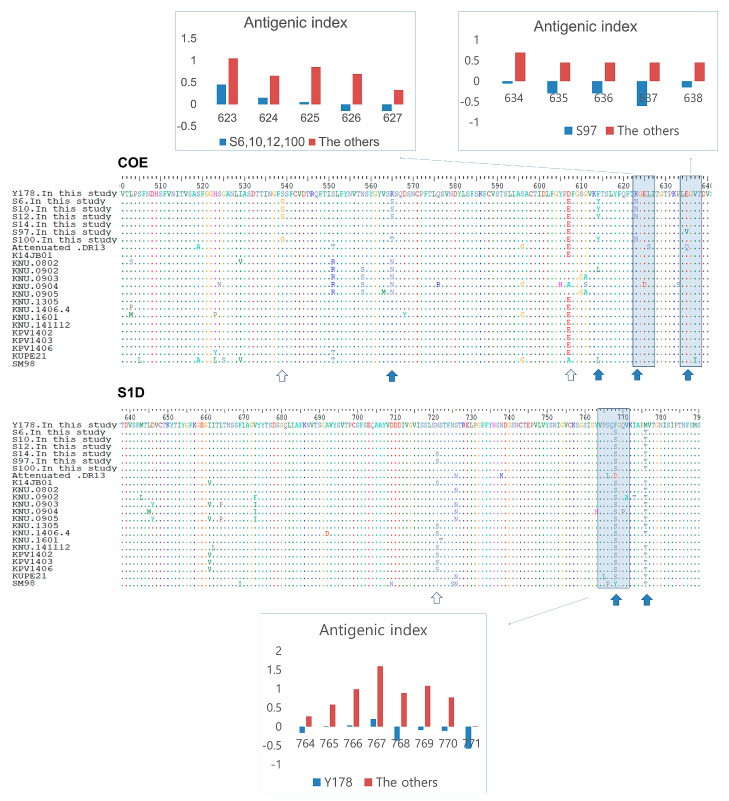
Antigenic index analysis of S protein B-cell epitopes in Korean *PEDV* strains. Strains S6, S10, S12, and S100 exhibited lower antigenic indexes, below the cut-off value of 0.5, at amino acids 623–627 of the COE region. Strain S97 exhibited reduced antigenic indexes, below 0.5, at amino acids 634–638 of the COE region. Strain Y178 exhibited reduced antigenic indexes, below 0.5, at amino acids 764–771 in the S1D region.

When analyzing the Korean *PEDV* strains—newly identified in this study—based on the three major B-cell epitope sequences of the S protein (COE, S1D, and 2C10), the six strains exhibited significantly lower antigenic indexes in some parts of the B-cell epitope sequences (Figure 6). The strains S6, S10, S12, and S100 exhibited lower antigenic indexes, below the cut-off value of 0.5, in amino acid positions 623–627 of the COE region, which contained the nonsynonymous change K623N. The S97 strain exhibited lower antigenic indexes, below the cut-off value of 0.5, in amino acid positions 634–638 in the COE region, and the nonsynonymous change E635V was observed in this region. The Y178 strain also showed antigenic indexes below 0.5 at amino acids 764–771 of the S1D region, which contained the nonsynonymous change S768F. It is likely that each of the mentioned nonsynonymous changes affected the antigenic indexes of the adjacent regions.

## 4. Discussion

First of all, this study reflected the ongoing circulation of *PEDV* in Korea at about 10% (Table 1) and its wide distribution (Figure 1). At the same time, obtaining genomic sequences of *PEDV* from field samples provided good opportunity for studying the genetic evolution of the virus. As a result, this study applied multiple bioinformatics tools to investigate the genetic diversity of *PEDV*. Based on phylogenetic analysis of the complete S gene or S gene excluding recombinant regions, global *PEDV* strains (Figure 2) can be classified into two genogroups, and each genogroup may be further subdivided into two (G1a and G1b) and three (G2a, G2b, and G2c) sub-genogroups. This result was consistent with that of Guo et al. [8]. Additionally, Guo et al. reported that all these different subgroups existed in Korea, with the most prevalent subgroup being G2a, when they analyzed the Korean *PEDV* strains identified prior to 2016. Similar to the previous data, all the Korean field strains identified from 2017 to 2018 in this study were included in subgroup G2a, indicating that the most prevalent Korean subgroup has not changed since 2016.

Genogroups G1 and G2 are known to have different S protein neutralization activities [16]. However, differences within the genogroups have not been investigated. The six Korean strains (S6, S10, S12, S97, S100, and Y178) had significantly reduced antigenic indexes compared with other Korean strains in some parts of the COE [40] and S1D [41], which encode the B-cell epitopes of the S protein. These antigenic index reductions may induce somewhat different S protein antigenicities, even within the same genogroup. In fact, strains S10, S12, S97, and S100 originated from swine farms that had suckling piglet mortalities of almost 100%. These swine farms had been regularly using killed vaccines containing the new genogroup (G2) *PEDV* strain but were seriously damaged by porcine epidemic diarrhea.

In the literature, the N gene had been used to infer the phylogenetic relationships of *PEDV* strains [44,45]. It was noteworthy that the N gene showed a similar evolutionary rate to the S gene in this study. This high evolutionary rate implies that the N gene, as well as the S gene, is likely to have high genetic diversity; accordingly, several sub-genogroups could have diverged. Comparing to the S-based phylogenetic topology, the classical strains (G1 strains) presented the same topology in the N-based and S-based analysis. However, there were some differences in the classification of subgroups on the new genotype strains (G2 strains). The N3 genogroup consisting of the G2 strains was divided into only 2 subgroups (N3a and N3b) not following the S-based subgroups (G2a, G2b and G2c). Specifically, the N3b strains were consistent with the G2b strains, but the G2a and G2c strains were grouped into the same subgroup, N3a, in the N-based topology. This classification of the three genogroups (N1, N2, and N3) was also supported by the consistent variation in the antigenic indexes depending on the genogroups. The number of antigenic indexes of the N3 strains significantly decreased compared to those of the N1 and N2 strains within amino acids 122–126, which code for the B-cell epitope of the N protein [43].

The *PEDV* N protein has an important immunological aspect. Abundant antibodies against the N protein are induced at the early stages of *PEDV* infection [12,13]. Furthermore, the N protein is considered to play an important role in inducing cell-mediated immunity [13]. As a result of these features, the N protein is commonly used as a target for diagnosis and vaccine development [10,46]. As mentioned above, several geno- and sub-genogroups based on the N gene were identified in this study. This genetic diversity may change their antigenicities according to their geno- or sub-genogroup (Figure 5). In the immunological diagnosis of *PEDV*, commercial *PEDV* ELISA kits have been showing poor performance, and the results of neutralizing assays using cell culture sometimes mismatch with those of the ELISA assays. In fact, Chang et al. recently reported that the antibodies induced by the G2b *PEDV* strain poorly reacted with a commercial N-based ELISA kit that showed a sensitivity of 37% [47], which may be the result of antigenicity differences between the genogroups. However, further study is required to validate this hypothesis. Indeed, if there are antigenic differences between the genogroups, a combination of N proteins derived from both genogroups would be required for the accurate immunological diagnosis of *PEDV*.

## 5. Conclusions

Overall, this study revealed that *PEDV* displayed genetic diversity in both the S and N genes, which resulted in the divergence into different sub-genogroups and altered antigenic indexes. Such *PEDV* mutants derived from genetic mutations of the S and N genes may cause severe damage to swine farms because of their ability to evade the unprepared host immune systems.

## Figures and Tables

**Table 1:** Information of *PEDV* positive samples in this study.

Sample Number	Sample Collection *			Farm Information			Other Pathogens *** (TGEV, RotaV, PDCoV, PRRSV, PPV, JEV)	GenBank
	Stage	Collected Date	Province	Farm Name	No. of Sows	Vaccinated **		
Y178	Suckling	21-Feb-2017	Gyeongnam	HA	1500	PRRSV (MLV), PPV (VLP)	Negative	MH891588
S6	Suckling	09-Jan-2018	Chungnam	DB	200	PRRSV (MLV), PPV (VLP)	Negative	MH891589
S10	Suckling	06-Feb-2018	Gyeonggido	WD	200	PPV (VLP), PRRSV (MLV), PEDV (killed)	Negative	MH891590
S12	Suckling	06-Feb-2018	Gyeonggido	WT	200	PPV (VLP), PRRSV (MLV), PEDV (killed)	Negative	MH891584
S14	Suckling	07-Feb-2018	Gyeonggido	SW	400	PPV (VLP), PRRSV (MLV), JEV (MLV)	Negative	MH891585
S97	Suckling	17-Apr-2018	Gyeonggido	NG	400	PPV (VLP), PRRSV (MLV), PEDV (Killed)	Negative	MH891586
S100	Suckling	19-Apr-2018	Gyeonggido	YG	700	PPV (VLP), PRRSV (MLV), JEV (MLV), PEDV (Killed)	Negative	MH891587

* Suckling piglets in the investigated farms suffered from severe watery diarrhea and high mortality. ** MLV: modified live vaccine, VLP: virus-like particle. *** *TGEV (Transmissible gastroenteritis virus), RotaV (Rotavirus group A), PDCoV (Porcine deltacoronavirus), PEDV (Porcine epidemic diarrhea virus), PRRSV (Porcine reproductive and respiratory syndrome virus), PPV (Porcine parvovirus), JEV (Japanese encephalitis virus)*.

**Table 2:** Detection results of *PEDV* according to each stage from 2017 to 2018.

2017 Year/Stage *	Suckling	Weaned	Growing	Finishing	Gilt	Sow	Total
Number of samples	325	162	42	37	43	63	672
Positive samples	33	15	2	3	2	3	58
%	10.15	9.26	4.76	8.11	4.65	4.76	8.63
2018 year/stage *	Suckling	Weaned	Grower	Finisher	Gilt	Sow	Total
Number of samples	76	58	34	20	18	29	235
Positive samples	16	8	2	2	2	2	32
%	21.05	13.79	5.88	10.00	11.11	6.90	13.62
Total/stage *	Suckling	Weaned	Grower	Finisher	Gilt	Sow	Total
Number of samples	401	220	76	57	61	92	907
Positive samples	49	23	4	5	4	5	90
%	12.22	10.45	5.26	8.77	6.56	5.43	9.92

* Samples were sorted into 6 stages: female (gilt and sow), suckling (<21 d), weaned (21–60 d), growing (60–90 d); and finishing (≥90 d).

**Table 3:** Estimated nucleotide substitution rates for the S and N genes.

Dataset	Data Best Fit Molecular Clock *	Data Best Fit Tree Prior	Geometric Mean Rate (×10^−4^) **	95% HPD Interval (×10^−4^) ***
D1: Complete S gene	RLC	Constant	5.23	3.81–6.53
D2: S gene without recombinant regions	RLC	Exponential	7.98	6.18–10.02
D3: Complete N gene	RLC	Constant	6.58	4.34–9.03
D4: N gene without recombinant regions	RLC	BSP	3.24	2.12–4.52

* Random local clock model (RLC). ** The geometric mean nucleotide substitution rate (substitutions/site/year) was inferred from the data that best fit the molecular clock and coalescent tree prior of constant population size, exponential population size, and Bayesian skyline plot (BSP). *** Highest posterior density (HPD).

## Supplementary Materials

The following are available online at https://www.mdpi.com/1999-4915/12/8/790/s1, Figure S1: Supplementary material-Figure-S1-S-nu-al-BMT-RLC-Const. Figure S2: Supplementary material-Figure-S2-S-nu-no-rec-regions-BMT-RLC-Expo. Figure S3: Supplementary material-Figure-S3-N-nu-al-BMT-RLC-Const, Figure S4: Supplementary material-Figure-S4-N-nu-no-rec-regions-BMT-RLC-BSP. Figure S5: Supplementary material-Figure-S5-N-substitutions-mapping-tree. Table S1: Supplementary-material-Table-S1-model-comparison-1. Table S2: Supplementary-material-Table-S2-model-comparison-2.
